# Entomological risk assessment for transmission of arboviral diseases by *Aedes* mosquitoes in a domestic and forest site in Accra, Ghana

**DOI:** 10.1371/journal.pone.0295390

**Published:** 2023-12-07

**Authors:** Nukunu Etornam Akyea-Bobi, Jewelna Akorli, Millicent Opoku, Samuel Sowah Akporh, Godwin Kwame Amlalo, Joseph Harold Nyarko Osei, Kwadwo Kyereme Frempong, Sellase Pi-Bansa, Helena Anokyewaa Boakye, Mufeez Abudu, Esinam Abla Akorli, Dominic Acquah-Baidoo, Rebecca Pwalia, Joseph Humphrey Kofi Bonney, Reginald Quansah, Samuel Kweku Dadzie

**Affiliations:** 1 Department of Parasitology, Noguchi Memorial Institute for Medical Research, University of Ghana, Legon, Accra; 2 Vestergaard NMIMR Vector Labs, Department of Parasitology, Noguchi Memorial Institute for Medical Research, University of Ghana, Legon, Accra; 3 Department of Virology, Noguchi Memorial Institute for Medical Research, University of Ghana, Legon, Accra; 4 Department of Biological, Environmental and Occupational Health, School of Public Health, University of Ghana, Legon, Accra; CEA, FRANCE

## Abstract

Dengue, Zika and chikungunya are *Aedes*-borne viral diseases that have become great global health concerns in the past years. Several countries in Africa have reported outbreaks of these diseases and despite Ghana sharing borders with some of these countries, such outbreaks are yet to be detected. Viral RNA and antibodies against dengue serotype-2 have recently been reported among individuals in some localities in the regional capital of Ghana. This is an indication of a possible silent transmission ongoing in the population. This study, therefore, investigated the entomological transmission risk of dengue, Zika and chikungunya viruses in a forest and domestic population in the Greater Accra Region, Ghana. All stages of the *Aedes* mosquito (egg, larvae, pupae and adults) were collected around homes and in the forest area for estimation of risk indices. All eggs were hatched and reared to larvae or adults for morphological identification together with larvae and adults collected from the field. The forest population had higher species richness with 7 *Aedes* species. The predominant species of *Aedes* mosquitoes identified from both sites was *Aedes aegypti* (98%). *Aedes albopictus*, an important arbovirus vector, was identified only in the peri-domestic population at a prevalence of 1.5%, significantly higher than previously reported. All risk indices were above the WHO threshold except the House Index for the domestic site which was moderate (19.8). The forest population recorded higher Positive Ovitrap (34.2% vs 26.6%) and Container (67.9% vs 36.8%) Indices than the peri-domestic population. Although none of the mosquito pools showed the presence of dengue, chikungunya or Zika viruses, all entomological risk indicators showed that both sites had a high potential arboviral disease transmission risk should any of these viruses be introduced. Continuous surveillance is recommended in these and other sites in the Metropolis to properly map transmission risk areas to inform outbreak preparedness strategies.

## Introduction

Dengue, Zika and chikungunya viruses, transmitted by *Aedes* mosquitoes of the subgenus *Stegomyia*, are usually reported in urban and peri-urban areas. All three viruses are originally transmitted in zoonotic cycles involving primates and mosquitoes [1]. However, human activities such as the domestication of the natural habitats of the primate reservoirs and vectors have led to an urban transmission cycle involving human-to-human transmission where *Aedes aegypti* and *Aedes albopictus* are major vectors [2, 3]. *Aedes* mosquitoes are highly invasive species and widespread on almost all continents [4]. *Aedes aegypti* is mainly found in the tropics and subtropics, flourishing in domesticated environments and has adapted to feeding almost solely on humans. It is active during the day and usually bites several people during the acquisition of a bloodmeal. These behavioural characteristics combined with its high susceptibility to the dengue, Zika and chikungunya viruses (DENV, ZIKV and CHKV) make it a very efficient vector [5]. *Aedes albopictus*, originating from Southeast Asia, is a highly invasive mosquito species that has spread rapidly over the last few decades [6, 7]. The spread of this vector is of concern due to its ability to spread arboviruses such as DENV, ZIKV and CHKV which are diseases of great public health importance [8]. This mosquito also prefers domesticated environments and has adapted to surviving in different types of climates.

Ecological and human factors seem to be strong determinants for the spread of arboviruses and their vectors [9]. The biology of the vectors, their abundance, and geographical distribution are directly affected by climate [10]. Global trade such as the importation of used tyres, ornamental plants and used vehicles is also one of the driving factors for the spread of *Aedes* mosquitoes [11]. Infected travellers also enable the virus to reach new places within the shortest possible time [12]. This can lead to an epidemic in areas where there is a well-established vector population. Rapid urbanization has also been documented as one of the drivers of the spread of arboviral diseases [13]. Most outbreaks of arboviral diseases in Africa have occurred in urban cities [14]. Poor planning of urban cities leading to issues like lack of consistent water supply, poor drainage and waste disposal systems facilitate the development of vectors [15].

In Africa, thirty-four countries have reported dengue outbreaks with their capital cities being the hardest hit [16]. Ghana shares borders with Burkina Faso, Cote d’Ivoire and Togo which have reported DENV, CHKV and ZIKV. However, several studies conducted in Ghana over the years, did not detect any outbreaks of these diseases despite the presence of *Aedes aegypti* mosquitoes in most parts of the country [17–19]. There is now evidence of DENV-2 (dengue virus serotype-2) antibodies in persons visiting the hospital with acute fever symptoms [20, 21]. DENV-2 viral RNA was also detected among two children in some localities in urban Accra while the patients had had no prior travel history outside of the country. This suggests that the infections were acquired locally and the virus is silently circulating in the population [22], calling for extensive studies that include investigating vector populations and their transmission risk potential. This study was aimed at determining the species composition, abundance, and distribution of *Aedes* mosquitoes and estimating the entomological risk for transmission of viral haemorrhagic fevers (VHF) and other arboviruses within a forest and a peri-urban community in Accra, Ghana, by comparing risk indices to the WHO VHF thresholds [23]. The study serves as a pilot towards designing comprehensive population assessment across the Metropolis. The two sites were selected to provide data to assess the risk in a sylvatic and domestic area and, identify where more attention may be required in risk mitigation programmes.

## Materials and methods

### Study area

**Fig 1:**
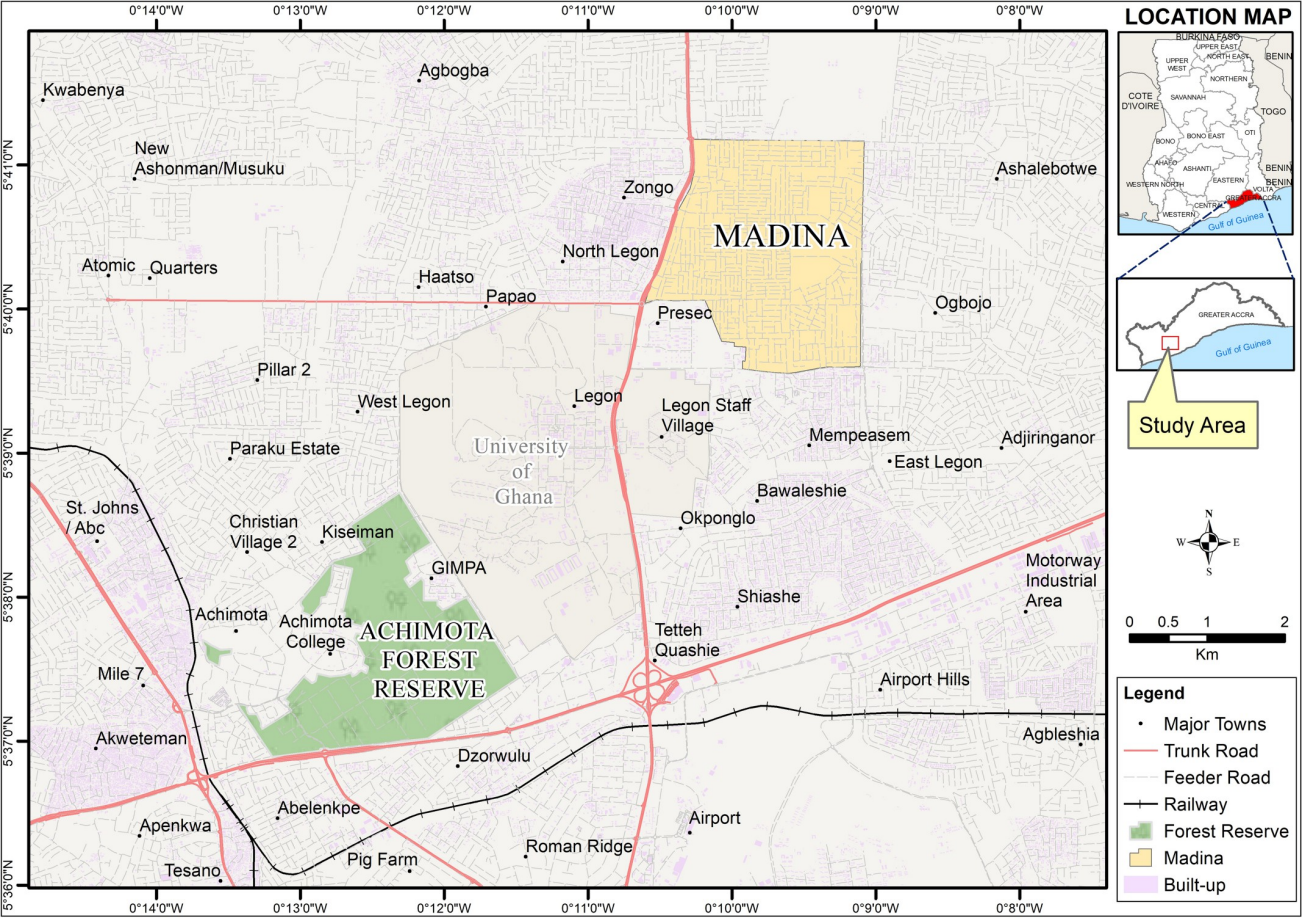
Map of Greater Accra region showing point locations of Achimota Eco-park and Madina. Maps were created with QGIS version 3.28.2-Firenze.

The study was carried out in two areas: Achimota Eco-park (urban forest area) and Madina (peri-urban community) located in Accra Metropolitan District of the Greater Accra Region, Ghana (Fig 1). Both areas are about 10.4km apart. Madina is the capital of the La-Nkwantanag-Madina Municipal, and covers a total area of 6.9km^2^. It is a peri-urban area which has over several years developed into a central business district and a hub for commercial activities. This has led to the influx of rural-urban migrants looking for better prospects. Vehicle repair shops and tyre sale shops are common businesses found within this community. These car spare parts and tyres found in these shops are imported from all over the world. Due to vertical transmission of these viruses, eggs attached to these substrates could potentially be carrying DENV, ZIKV and CHIKV [24], presenting the possibility of introducing *Aedes* mosquitoes carrying arboviruses into the community.

Achimota is a 3.6km^2^ eco-tourist site which also hosts the Accra Zoo. People visit the forest eco-park from various parts of the country for other recreational and religious activities. The presence of these patrons in the forest area where there are animals that serve as reservoirs for these viruses makes it a probable site for sylvatic-human or human-sylvatic arboviral transmission.

### Ethical statement

This study was part of a larger *Aedes* and arboviral surveillance project conducted at Noguchi Memorial Institute for Medical Research (NMIMR), University of Ghana under ethical approval (033/15-16) provided by the Noguchi Institutional Ethical Review Board (Federal Assurance #: 00001824). Permits to set traps to collect samples in the peri-domestic sites was not required. However, verbal consent was obtained from household owners to set traps close to their homes. To collect in the forest, permission was obtained from the forestry commission. Forest guards escorted us during the setting and retrieval of traps.

### Mosquito collection

*Aedes* mosquito eggs and larval sampling was conducted between July-September 2020. Using the KoBoCollect and Geographical Positioning System (GPS) Essentials app, the geographical co-ordinates of the point within the study area where the various stages of the *Aedes* mosquito were sampled, were recorded.

Ovitraps to collect *Aedes* eggs were prepared from plastic water bottles cut into two halves and painted black on the outside. Each trap was lined with brown paper and filled with approximately 300mL of hay-infused water. Each study area was divided into four quadrants and in each quadrant, 10 ovitraps were placed randomly, at least 80m apart. The ovitraps were left for 5 days, retrieved and replaced over 3 different time points (~2 weeks apart) within the study period. During retrieval, the brown paper lining was checked for the presence of eggs, removed from the trap and stored in labelled Ziploc bags. The hay-infused water was also checked for larvae, and if any, was poured into collection bags and returned to the lab. The number of eggs per trap was counted with the aid of a microscope. Brown paper containing eggs and the water contents of the ovitraps were poured into larval trays in the laboratory and allowed to hatch. Larvae were identified using morphological identification keys [25].

Water-holding receptacles which are potential breeding sites were also inspected for *Aedes* larvae and pupae to enable estimations for larval risk indices. In Madina, houses within the quadrants were visited to seek permission to conduct larvae and pupae sampling. We initially planned for 20 houses per quadrant, but our period of sampling was a few months post COVID-19 lockdown, so inhabitants were not very receptive to having us in their homes. We therefore visited as many houses as we could within the study area (without aiming to have an equal number of houses per quadrant) to achieve our number of required consenting homes. Verbal consent was obtained from inhabitants to inspect water storage containers that were placed on their porches and balconies. In addition, the compounds and immediate environments outside each house (peri-domestic) were also scouted for receptacles such as flowerpots, disposed cans, bottles and tyres that could have *Aedes* larvae and pupae. In the forest study area discarded cans, car tyres were also inspected for *Aedes*. The total number of inspected receptacles, positive containers, and houses where *Aedes* was found were recorded. As the focus of the study was not to identify the most conducive container for *Aedes* breeding, the larvae and pupae collected were pooled for each study area and transported to the laboratory for identification.

The Biogents-sentinel traps (BG-traps) with BG lure were set to capture adult *Aedes* mosquitoes outdoors. Using the previous quadrant demarcations, a BG trap was placed per quadrant outside a randomly selected house or property, after obtaining permission from the owner. The collection was done over a period of 12 hours (6:00 am to 6:00 pm). Traps were set 2 days a week per quadrant over a 3-week period, each time changing the house where the BG traps were placed. The adult mosquitoes caught by the trap were collected into sealed vials, transported to the laboratory, identified and stored at -80°C for further analyses.

### Morphological identification of *Aedes* mosquitoes

All *Aedes* mosquitoes collected from the field were morphologically identified using identification keys [25] and, with the aid of a stereomicroscope at total magnifications of 40x and 20x. After identification, the mosquitoes were grouped into pools of 20 for the larvae and groups of 10 for the adults according to species and preserved in RNALater (SIGMA®) at -80°C for viral detection.

### Viral detection using Trioplex Real-time RT-PCR assay

Each sample was homogenized in Minimum Essential Medium (MEM) containing Earle’s Salts, 2% L-glutamine, supplemented with 10% heat inactivated FBS and Penicillin-Streptomycin. The Qiagen Viral RNA Mini Kit (Qiagen, Hilden, Germany) was used according to the manufacturer’s instructions to extract viral RNA from pools of mosquitoes. The AgPath-ID RT-PCR kit was used for detection of DENV, CHIKV and ZIKV according to an established protocol [26]. The kit came with a positive control set (CDC; catalog #KT0167) which included DENV Positive Control: Inactivated dengue virus, CHIKV Positive Control: Inactivated chikungunya virus, ZIKV Positive Control: Inactivated Zika virus, Human Specimen Control (HSC): extraction control and positive control. Molecular grade nuclease free water was used as no-template control.

### Data analyses

The relative abundance of different species of *Aedes* mosquitoes were calculated per site. Three larval indices; House index (HI- the percentage of houses infested with *Aedes* larvae or pupae), Container Index (CI- the percentage of containers infested with larvae or pupae) and Breteau index (BI- the number of positive containers per 100 inspected houses) were used to estimate mosquito density risk in each study sites. Positive ovitrap Index (POI- percentage of ovitraps positive with *Aedes* eggs) and Egg Density Index (EDI–average number of eggs per traps evaluated) were used to assess mosquito oviposition activity. All indices estimated were compared to WHO thresholds for transmission risk of viral haemorrhagic fevers (VHFs) [23].

## Results

### Species abundance and composition of *Aedes* mosquitoes

**Fig 2:**
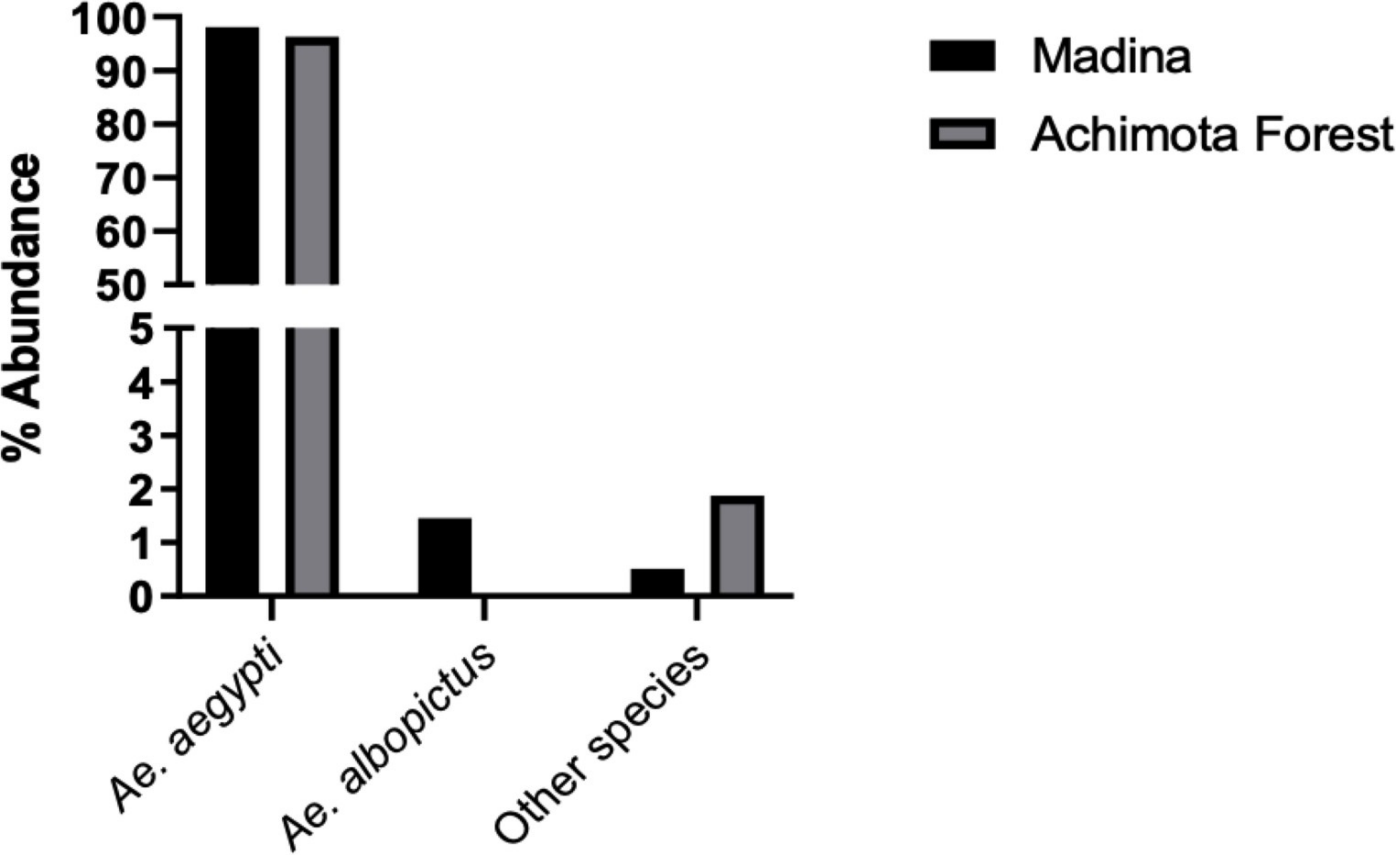
Species composition and abundance of *Aedes* mosquitoes identified from Achimota forest and Madina.

A total of 2218 mosquitoes, which included BG-trapped adults (Achimota = 8; Madina = 28), larvae reared from eggs and larvae collected from containers, were identified to the species level (Fig 2, S1 File: S1 Table). The most common species of *Aedes* mosquitoes identified was *Aedes aegypti* which represented 98% of all mosquitoes collected from both study areas. Other species identified in Madina included *Aedes albopictus* (1.5%), *Aedes simpsoni* (0.2%) and *Aedes ingrami* (0.3%). Achimota forest recorded a higher *Aedes* species richness and recorded *Aedes ingrami* (0.4%), *Aedes simpsoni* (0.3%), *Aedes domesticus* (0.4%), *Aedes africanus* (0.7%), *Aedes vittatus* (0.1%) and *Aedes de-boeri* (0.1%).

### Ovitrap indices

A total number of 120 ovitraps were set in each study area but 106 traps were retrieved per site. Unretrieved traps were either destroyed or missing. The total number of traps positive for *Aedes* eggs in Achimota forest and Madina were 38 (35.9%) and 29 (27.4%), respectively (Fisher’s exact: *P* = 0.25, 95% CI = 0.796–2.777). The total number of eggs counted from the positive ovitraps were higher in Achimota forest (1614) than in Madina (828), representing 42.5 and 28.5 average egg count per positive ovitrap, respectively. Similarly, the calculated egg density per trap (average number of eggs per traps retrieved) was 15.2 in Achimota forest and 7.8 in Madina, also implicating the low availability of conducive breeding spots in the forest area. The few available sites, such as an ovitrap, are therefore probably visited by many gravid females to lay eggs in the forest.

### Larval indices

Similar larval counts were realised from Madina (560) and Achimota (590) during the larval survey. A total of 81 houses were inspected in Madina for *Aedes* larvae and, 16 (19.8%) were positive. No houses were surveyed in Achimota forest because there were no living quarters available. A total of 155 outdoor or peri-domestic containers were also inspected in Madina and 57 (36.8%) were positive. In Achimota forest, 53 containers were inspected and 36 (67.9%) were found positive for *Aedes* larvae. These results indicate that chances of finding larvae in containers in the forest are higher than observed in the peri-domestic environment close to human settlement (Fisher’s exact: *P* = 0.0001, 95% CI = 0.133 0.557). Some examples of containers and breeding sites that were inspected included plastic and metal barrels, buckets, earthenware pots, Polytanks® (large water storage tanks), abandoned bathtubs, abandoned machine parts and car tyres. Tyres were the most positive containers for *Aedes* larvae and pupae in both sites (S1 File: S2 Table).

### Comparison of entomological risk indices

Entomological risk indices estimated in this study were compared to WHO VHF transmission risk threshold values (Table 1). All estimated indices were indicative of high VHF transmission risk. The Breteau and Container indices were 16–20 above adequate risk of transmission, while the House index showed moderate risk.

**Table 1:** Larval and ovitrap indices for risk of transmission of *Aedes* -borne viral diseases in Madina and Achimota Forest.

Entomological indices for study areas	Madina	Achimota Forest	WHO threshold for transmission risk of VHF’s
**Egg density index**	7.8	15.2	NA
**Positive ovitrap index**	27.4*	35.5*	> 10
**Container index**	36.8*	67.9*	3–20 or above
**House index**	19.8*	NA	4–35 or above
**Breteau index**	70.4*	NA	5–50 or above

(*) indicates areas with high VHF transmission risk

### Viral detection

A total of 120 mosquito pools was tested for the presence of DENV, ZIKV and CHKV. These consisted of a total of 1493 *Aedes aegypti* mosquitoes (larvae and adults) and 1 pool of *Aedes albopictus*. All the pools tested negative for DENV, ZIKV and CHKV.

## Discussion

The presence of *Aedes* mosquitoes has been well documented in Ghana and, *Ae*. *aegypti* is a vector species of yellow fever in the country [17, 18]. Studies conducted in 2016 and 2018 have detected the presence of antibodies and viral RNA of DENV in Cape Coast and Accra, Ghana [20, 21]. This has emphasized the need to investigate and understand the role of *Aedes* populations in the spread of these diseases in the country, which will inform the formulation of effective and efficient strategies to prevent future outbreaks or mitigate any outbreak that may arise. This study focused on measuring the risk of transmission of *Aedes*-borne viral diseases, specifically DENV, ZIKV and CHKV in a domestic (Madina) area and in a forest area (Achimota forest). The most abundant species of the *Aedes* mosquito recorded in both populations was *Aedes aegypti*, consistent with other studies conducted in Ghana [19, 27]. The high abundance of these vectors in the country have been attributed to the presence of water holding containers close to human dwellings serving as breeding sites for these mosquitoes. *Aedes aegypti* has over the years evolved to live close to humans and preferentially choose to feed almost solely on humans even in the presence of other vertebrates that could serve as host [28]. Therefore, the availability of breeding sites close to human dwellings may lead to a rise in the population of these *Aedes* species. We observed the proximity of positive breeding sites to houses in Madina, providing evidence for the high abundance of the *Aedes aegypti* vector. This characteristic of *Aedes aegypti* increases the potential for an outbreak should the virus be introduced into these areas [29].

One key finding in this study was the detection of *Aedes albopictus* in Madina. The presence of this vector has been documented before in Ghana [18], but our results indicate an increased prevalence. These mosquitoes are highly invasive species and numbers such as these could be an indication of a well-established population of *Aedes albopictus* mosquitoes locally. We only observed *Aedes albopictus* in the larval stages during the surveys; no adults were collected. Unfortunately, our sampling strategy did not allow designation of these larvae to their respective breeding containers, or state how many receptacles were positive for these species. *Aedes albopictus* is another competent vector of DENV, CHKV and ZIKV and the presence of two competent vectors of these viruses in Madina puts the community at an even greater risk for the transmission should these viruses be introduced into the population [30–32]. We also identified *Aedes vittatus*, principal forest and savannah species, in the Achimota forest. In some parts of Africa, *Aedes vittatus* has been linked to the spread of yellow fever and has also been experimentally proven to spread ZIKV [33–35].

Water holding containers in and around homes were inspected for the presence of the aquatic stages of the *Aedes* mosquito to allow for the estimation of the larval risk indices. Both study sites recorded values above the WHO threshold except the House index for Madina which fell within the moderate risk range. These high values observed indicate a high risk of transmission should the dengue, Zika and chikungunya viruses be introduced into these communities through the presence of a positive human host, reservoir, or vector. The relatively lower value observed for the House index in Madina could be attributed to observed conditions of the Polytanks® (large water storage containers) and plastic barrels for storage of water used in the community. Most water storage containers were either empty or were properly covered if they had water in them. This prevented mosquitoes from gaining access to the water within to lay eggs. The community also has regular supply of water therefore there is little need for water storage. These observations are important *Aedes* control strategies that must be strongly emphasised. Regular supply of water is essential to avoid water storage in receptacles. However, in areas where regular water supply through the taps is scarce, storage containers must be tightly covered.

Most containers found positive were observed in water holding containers outside of human dwellings such as discarded bicycle and car tyres, and cans. Similarly, a high number of tyres discarded near the forest office buildings were also positive for *Aedes* mosquitoes, confirming findings from other studies that discarded tyres have high, if not the highest, positivity rate for *Aedes* larvae compared to other water holding containers [18, 27]. Water collected in these tyres are left undisturbed for long periods of time. In addition to the dark colour, tyres provide cool and shady environments conducive for *Aedes* mosquito development [36].

None of the mosquitoes was found to harbour DENV, ZIKV and CHIKV, similar to other reports in Ghana [17, 18]. It has been suggested that *Aedes aegypti* mosquitoes originating from the African continent may be less competent vectors of these viruses [37, 38]. The ability of these mosquitoes to serve as competent vectors is influenced by several biotic and abiotic factors. Some of these viruses are extremely vector specific and biotic factors such as the bacterial communities associated with the vectors may interfere with the ability of the virus to replicate or be transmitted [39–41]. It has also been reported that Ghanaian strains of *Aedes aegypti* were refractory to infection by DENV-serotype 2 [42]. Considering that there have been no reported outbreaks of DENV, ZIKV and CHIKV, relative higher numbers of mosquitoes are required to discover these infections in the vector.

## Conclusions

The *Aedes* mosquito populations in Ghana are competent vectors of yellow fever and, the indices estimated in this study reiterates the high risk they may potentially pose to transmission of other arboviruses. Relatively high numbers of *Aedes albopictus* detected in Madina from when a similar study was conducted in 2016 could signify the establishment of a local population. The detection of *Aedes vittatus* also raises concern since it has been implicated in the transmission of yellow fever and has been proven experimentally to transmit ZIKV. These confirm the need for a surveillance programme to regularly monitor the species abundance, diversity, and risk of transmission of arboviral diseases in the country and, include screening vectors for the presence of viral infections. There are, however, several factors and avenues that still need to be studied to fully understand the population structure and vector competence of *Aedes*, and the role of primate reservoirs in arboviral transmission in Ghana.

## Supporting information

S1 File Species diversity and abundance of mosquitoes collected from Madina and Achimota Forest (S1 Table) and container and ovitrap data in Madina and Achimota Forest (S2 Table). (DOCX)
