# Supplementary material for: Entomological risk assessment for transmission of arboviral diseases by Aedes mosquitoes in a domestic and forest site in Accra, Ghana
Source: PLoS One. 2023 Dec 7;18(12):e0295390. doi: 10.1371/journal.pone.0295390 (PMC10703219; doi:10.1371/journal.pone.0295390)
Supplement: S1 File — (DOCX) [file pone.0295390.s001.docx]

# S1 Table: Species diversity and abundance of mosquitoes collected from Madina and Achimota Forest

#

| Study site |  |  |  | *Aedes* species collected | |  |  |  |  |
| --- | --- | --- | --- | --- | --- | --- | --- | --- | --- |
|  | *Ae. aegypti*  N (%) | *Ae. albopictus*  N (%) | *Ae. Ingrami*  N (%) | *Ae. simpsoni*  N (%) | *Ae. Domesticus*  N (%) | *Ae. africanus*  N (%) | *Ae. vittatus*  N (%) | *Ae. de-boeri*  N (%) | Total |
| Madina | 1148  (98) | 17 (1.5) | 4  (0.3) | 2  (0.2) | 0 | 0 | 0 | 0 | 1171 |
| Achimota  Forest | 1027 (98.1) | 0 | 4  (0.4) | 3  (0.3) | 4  (0.4) | 7  (0.7) | 1  (0.1) | 1  (0.1) | 1047 |

Total 2175 17 8 5 4 7 1 1 2218

# S2 Table: Container and ovitrap data in Madina and Achimota Forest

|  | **Total number of ovitraps retrieved** | **Total number of positive ovitraps retrieved** | **Total number of eggs counted** | **Total number of containers inspected** | **Positive containers** | |
| --- | --- | --- | --- | --- | --- | --- |
|  |  |  |  |  | **Tyres**  N (%) | **Other containers**  N(%) |
| **Madina** | 106 | 29 | 828 | 155 | 27 (47%) | 30 (53%) |
| **Achimota Forest** | 106 | 38 | 1614 | 53 | 31 (86%) | 5 (14%) |

### 
